# The Lateral Breast Flap Sling: A Novel Technique for the Revision of Autologous Breast Reconstructions

**DOI:** 10.7759/cureus.10323

**Published:** 2020-09-09

**Authors:** Madison J Greer, Ivo A Pestana

**Affiliations:** 1 Plastic and Reconstructive Surgery, Wake Forest Baptist Medical Center, Winston-Salem, USA

**Keywords:** autologous breast reconstruction, breast reconstruction revision, breast revision, free flap, breast suspension

## Abstract

Introduction

A wide breast footprint is a common complaint expressed by breast reconstruction patients following abdominally-based autologous breast reconstruction. Our aim is to describe the lateral autologous breast sling, a novel technique, which modifies the lateral flap inset to address this common patient complaint.

Methods

A review of consecutive women who underwent the lateral autologous breast flap sling procedure over a four-year period was completed. Patient demographics, oncologic treatment, operative interventions, surgical indications, and complications were evaluated.

Results

Fourteen patients underwent 21 lateral autologous breast flap sling procedures with a mean follow-up of 18 months. Eleven patients underwent delayed breast reconstruction while three were completed immediately, including one patient who had concurrent unilateral mastectomy, free tissue transfer, and a lateral breast sling procedure. Revision surgery was sought for breast asymmetry, excessive lateral breast tissue, and poor superior pole volume. Simultaneous revision procedures were performed in 12 patients and included fat grafting, abdominal donor site revision, contralateral breast reduction, and V to Y advancement of the lateral breast/ chest soft tissues. Reconstruction was complete in 10 patients, with an average duration of 13.5 months and four reconstructive procedures. There were no major perioperative complications. Three patients developed fat necrosis following lipofilling and two of these patients required drainage and/or excision of fat necrosis.

Conclusions

The lateral autologous breast flap sling technique adds to the armamentarium for narrowing the wide breast and improving the lateral breast curve. It may be performed in combination with other revision procedures. The use of this technique is associated with a low complication rate and does not significantly increase the total number of reconstructive procedures or duration of reconstruction. This technique may be useful during initial free tissue transfer.

## Introduction

Breast reconstruction seeks to create a natural breast mound following the removal of the breast and the disruption of its anatomic boundaries. Once the breast mound and its footprint are established, the reconstructive surgeon can mold the breast’s subunits to achieve the ideal aesthetic outcome. The aesthetic subunits of the breast include three transition lines from the breast to the anterior axillary line, inframammary fold, and sternum [1]. Regardless of the type of breast reconstruction employed, patients frequently desire interventions that modify breast subunits to augment upper pole fullness and improve the appearance of these critical transition lines.

Patients who undergo abdominally-based free tissue transfer breast reconstruction often have a breast flap that is wide in its transverse dimension, resulting in excess lateral breast tissue and an unshapely lateral breast contour. Several techniques have been described to improve the lateral breast following this form of breast reconstruction. Most commonly, suction-assisted lipectomy of the lateral flap, dermatolipectomy of the lateral breast and chest soft tissues, and local tissue rearrangements have been utilized [2-3]. While these are effective options for reducing excess tissue of the lateral breast, they may produce flattening of the lateral breast and blunting of the lateral breast curve. In addition, these techniques do not contribute to the improvement of soft tissue deficits of the breast’s upper pole.

To address these patient complaints and the downsides of previously described techniques for lateral breast modifications, the lateral autologous breast sling technique has been developed. The technique consists of the release of the lateral aspect of the autologous flap, transposition of this portion of the flap to a more anterior and superior position, and securing it to the pectoralis major muscle along the anterior axillary line. We present the technique and case series of its use.

## Materials and methods

An internal review board-approved retrospective study, including consecutive patients who underwent the procedure over a four-year period, was completed. Women with abdominally-based autologous breast reconstruction who underwent the lateral autologous breast sling for improvement of the lateral breast contour, breast asymmetry, wide breast footprint, upper pole volume deficit, and/or breast ptosis were included in the study. Patients who had improvement of these complaints with techniques other than the lateral breast flap sling were not included in this review. Patient medical records were reviewed and data collected included patient demographics, oncologic treatments, operative procedures for breast reconstruction, including revision procedures and their complications. Statistical analysis included independent t-tests to examine group differences for continuous measures. Statistical significance of p<0.05 was utilized.

Technique

In all cases, breast mound creation with free tissue from the abdominal donor site was performed in the standard manner, utilizing the deep inferior epigastric pedicle as donor vessels and the internal mammary pedicle as recipients for microvascular anastomosis. Poorly perfused regions of the flaps were assessed by clinical examination or fluorescent angiography and resected. Flap inset (unilateral and bilateral breast reconstructions) was to the contralateral chest with the flap apex placed in the superior position in a vertical inset pattern to provide ideal pedicle and microvascular construct positioning and to maximize the position of well-perfused flap segments.

**Figure 1:**
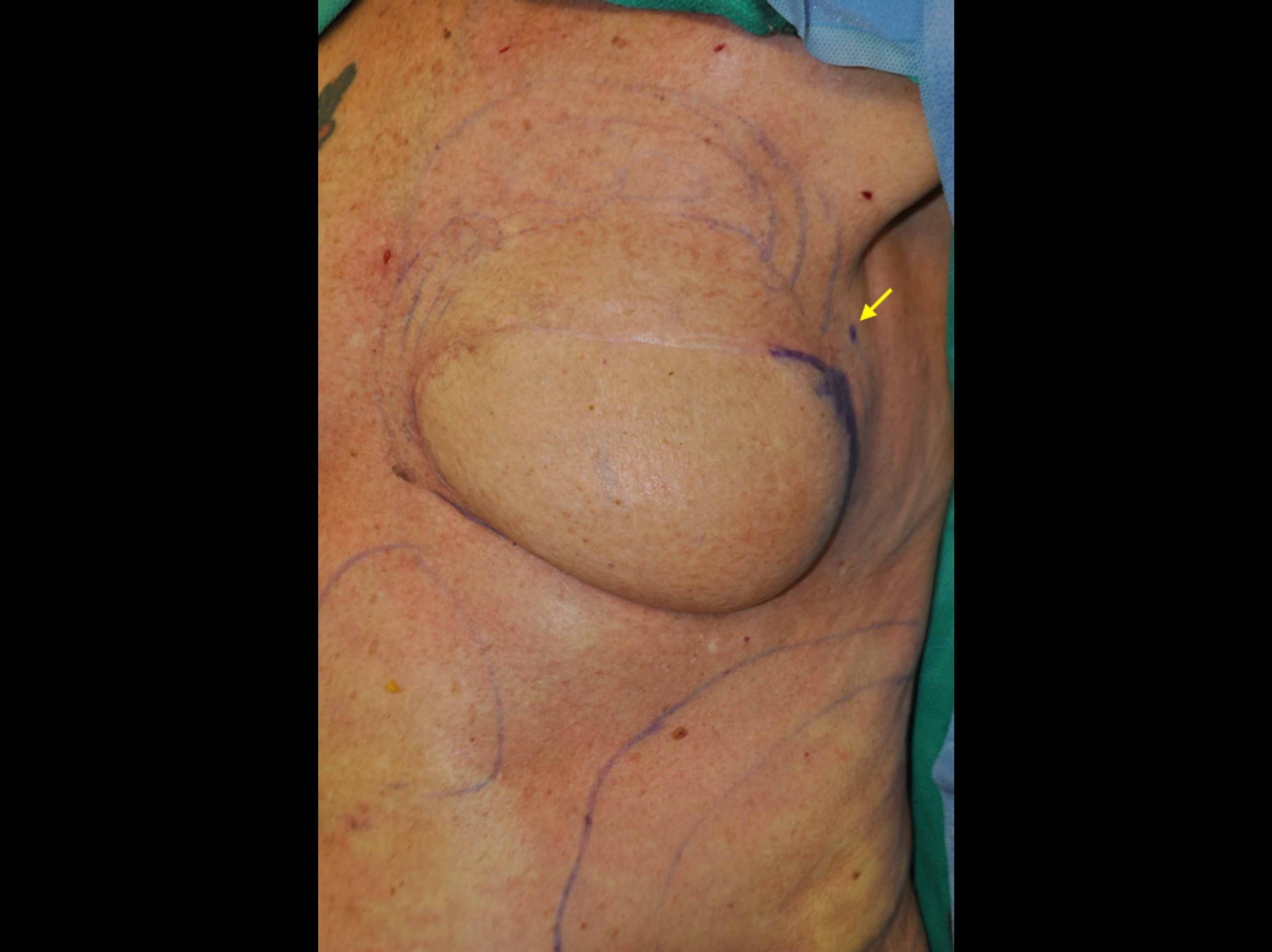
Preoperative markings demonstrating the planned lateral autologous flap incisions for release from the lateral chest wall soft tissues The superolateral dot (arrow) is the target for the elevation and suspension of the lateral flap to the pectoralis major. Circular marks of the abdomen and superior breast pole indicate planned fat graft harvest sites and lipofilling regions, respectively.

Markings for the lateral autologous breast sling procedure include the midline, planned lateral flap incisions used for the release of the flap, and critical transition lines of the breast, specifically the inframammary fold and the planned anchoring position to the pectoralis major (Figure 1). This anchor position is determined by the manual elevation of the lateral breast flap tissues to the anterior axillary fold and evaluation of the resultant lateral breast curve. If concurrent revision procedures are to be completed, their markings are completed as indicated. Fat grafting donor sites of the abdomen and flank and their recipient sites are marked.

The lateral inset of the breast flap is incised and the full thickness of the flap is dissected free from the native lateral chest soft tissues in the sub-flap plane. Dissection is carried medially to a level medial to the lateral margin of the pectoralis major. Once the lateral flap is adequately mobile and the lateral margin of the pectoralis major exposed, the patient is placed in the seated position and the apex of the released lateral portion of the flap is tucked under native chest soft tissues to the proposed anchor point to confirm the adequacy of breast narrowing, improvement of the lateral breast curve, and effect of flap repositioning on volume redistribution to the upper pole. Once confirmed, the released lateral flap apex is secured to the lateral edge of the pectoralis muscle utilizing two to four simple or figure-of-eight 2-0 Vicryl sutures. The resultant excess lateral chest skin is tailor tacked to the lateral aspect of the new lateral breast curve and buried flap skin is de-epithelialized in the standard fashion with care to maintain anchoring sutures. If needed, closure of the lateral breast may be closed in a V to Y fashion. The incision lines are closed in layers and the use of closed suction drains is optional (Video 1). 

**Video 1:** Compilation of intraoperative videos depicting skin marking and incisions, flap elevation, lateral flap mobility assessment, and suspension selection of released lateral breast flap The narrowed breast footprint is evaluated before the final suture suspension of the apex of the released flap to the pectoralis major musculature. After the suspension, the improved lateral breast curve and narrowed breast footprint are confirmed. The buried skin is then de-epithelialized and the flap re-inset.

## Results

Fourteen women underwent 21 lateral autologous breast flap sling procedures during the four-year study period. The characteristics of the included patients are listed in Table 1. The average patient age was 56 years (range 44-69 years) with an average body mass index (BMI) of 29 kg/m^2^ (range 23-37 kg/m^2^). The average follow-up time was 18 months (range 4-36 months). Prior to breast reconstruction, six patients were treated with chemotherapy and radiation therapy and one patient received adjuvant chemotherapy alone. Minimal active medical comorbidities were identified in the study group and the mean American Society of Anesthesiologist (ASA) physical status was 2.5.

**Table 1:** Patient characteristics

Total Patients, n	14
Age, Mean (range), years	56 (44-59)
Follow-up, Mean (range), months	18.1 (4-36)
Body Mass Index (BMI), Mean (SD), kg/m2	29 (3.8)
Normal Weight, n	2
Overweight, n	6
World Health Organization Obesity Class 1, n	5
World Health Organization Obesity Class 2, n	1
World Health Organization Obesity Class 3, n	0
American Society of Anesthesiologists (ASA) physical status, mean	2.5
Neoadjuvant or Adjuvant Chemotherapy, n, (%)	7 (50%)
Radiotherapy, n, (%)	6 (43%)
Diabetes, n, (%)	0 (0%)
History of Smoking, n, (%)	6 (43%)
Active Tobacco Use, n, (%)	0 (0%)
History of Deep Vein Thrombosis (DVT)/Pulmonary Embolus (PE), n, (%)	0 (0%)
Hypertension, n, (%)	3 (21%)

The operative characteristics of the included patients are delineated in Table 2. Nine patients underwent mastectomy for invasive ductal carcinoma (IDC), one patient for invasive lobular carcinoma (ILC), three patients for ductal carcinoma in situ (DCIS), and one patient for mixed pathology. Ten women underwent bilateral mastectomy and four patients underwent unilateral mastectomy. Eleven patients underwent delayed reconstruction and three patients had immediate breast reconstruction. Of the immediate reconstructions, one patient had concurrent unilateral mastectomy, free tissue transfer, and the lateral breast sling procedure. The abdominally-based flaps employed included all variations commonly used for breast reconstruction. Specifically, free transverse rectus abdominis myocutaneous flaps (fTRAMs), muscle-sparing free transverse rectus abdominis myocutaneous flaps (ms fTRAMs), and deep inferior epigastric perforator (DIEP) flaps were utilized.

**Table 2:** Operative Characteristics of the Study Cohort

Mastectomy Indication	
Invasive Ductal Carcinoma (IDC), n	9
Invasive Lobular Carcinoma (ILC), n	1
Ductal Carcinoma In Situ (DCIS), n	3
Mixed Pathology, n	1
Risk Reduction, n	0
Total Number of Breasts Reconstructed in the 14 Study Patients, n	24
Number of Patients With Immediate Reconstruction, n, (%)	3 (21%)
Number of Patients With Delayed Reconstruction, n, (%)	11 (79%)
Autologous Flap Type	
Free Transverse Rectus Abdominis Myocutaneous Flaps (fTRAM), n, (%)	7 (29%)
Muscle-Sparing fTRAM, n, (%)	7 (29%)
Deep Inferior Epigastric Perforator (DIEP), n, (%)	10 (42%)
Number of Lateral Autologous Flap Sling Procedures in the 14 Study Patients, n	21
Mean Time to Sling Performance Following Free Flap, Mean (Range), Months	5.9 (0-14.2)
Concomitant Procedures Performed With Sling	
Autologous Fat Transfer, n	11
Abdominal Scar Revision, n	7
Contralateral Symmetry Breast Reduction/ Mastopexy, n	1
Ipsilateral V-Y Tissue Rearrangement of Lateral Breast, n	1
Sling Procedure Perioperative Complications	
Flap Compromise/OR Takeback, n	0
Deep Vein Thrombosis (DVT)/ Pulmonary Embolus (PE), n	0
Hematoma, n	0
Seroma, n	0
Delayed Wound Healing, n	1
Insufficient Breast Correction, n	0
Fat Necrosis, n	3

Indications for the performance of the lateral breast flap sling included the lack of superior pole volume, excessive lateral breast tissue/wide breast, breast ptosis, and asymmetries. Twelve of the 14 patients included in the study underwent revision surgery with the lateral flap sling technique four to eight months after initial reconstruction. One patient underwent the lateral breast sling revision 14 months after initial reconstruction due to continued breast asymmetry despite V to Y tissue rearrangement and fat grafting of the reconstructed breast and contralateral breast reduction. The lateral breast sling was performed simultaneously with fat grafting in 11 patients and concurrently with abdominal dog-ear revision in seven patients. Four patients underwent additional breast revision procedures after the performance of the lateral breast sling, including additional fat grafting and scar revision two to four months following the breast sling procedure (Table 2).

**Figure 2:**
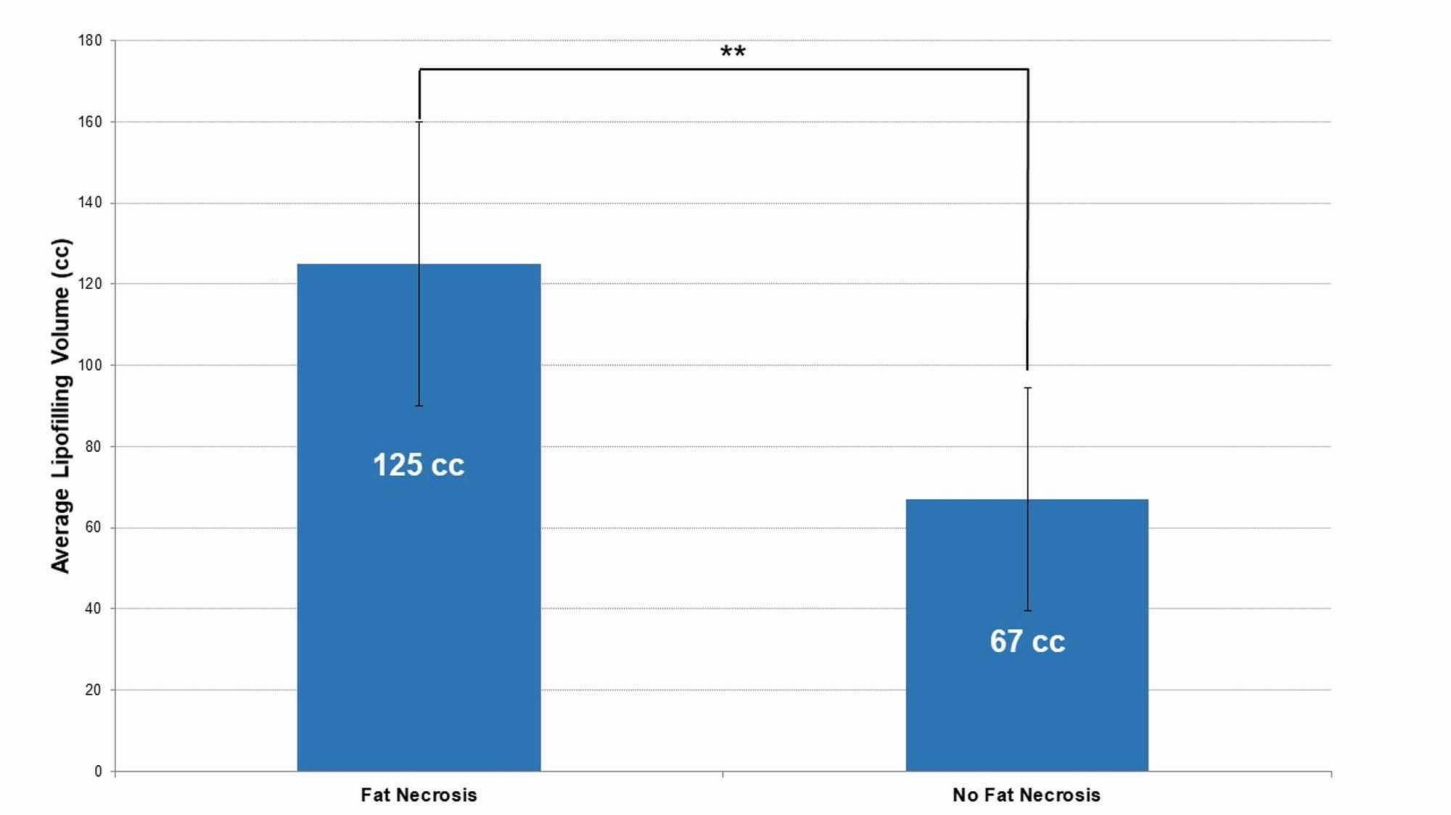
Comparison of fat graft volumes in patients who developed fat necrosis versus those who did not develop fat necrosis. The average volume of lipofilling per breast differed in patients that developed fat necrosis (n=3) versus those who did not develop fat necrosis after fat grafting (n=11). **Unpaired t-test, p = .0081.

Three of 14 women developed fat necrosis after the breast sling procedure and concurrent fat grafting. The average lipofilling volume was 125 cc per breast in those women compared to 67 cc in those who did not develop fat necrosis (Figure 2). All women who underwent fat grafting did so due to upper pole soft tissue deficits incompletely ameliorated by flap volume redistribution with the lateral breast sling procedure. Two of the three women who developed fat necrosis required interventions for the management of these sequelae of fat grafting. One patient developed left breast erythema and fluid collection two months after concurrent fat grafting and breast flap sling. She required drainage of the collection by interventional radiology but no bacteria were identified on gram stain or culture. One month later, this same patient developed a 3 x 3 cm indurated mass that was excised and pathology indicated fat necrosis. The second patient developed breast erythema and fluid collection one month after fat grafting and breast sling surgery. She underwent drainage of the collection in the clinic, which was consistent with fat necrosis. Packing of the drained region was initiated, and the patient subsequently underwent uneventful delayed-primary closure. One additional patient developed upper pole firmness suspected to be fat necrosis, but this resolved without intervention. No additional wound healing or perioperative complications occurred in the cohort of patients who underwent the lateral breast sling procedure.

**Figure 3:**
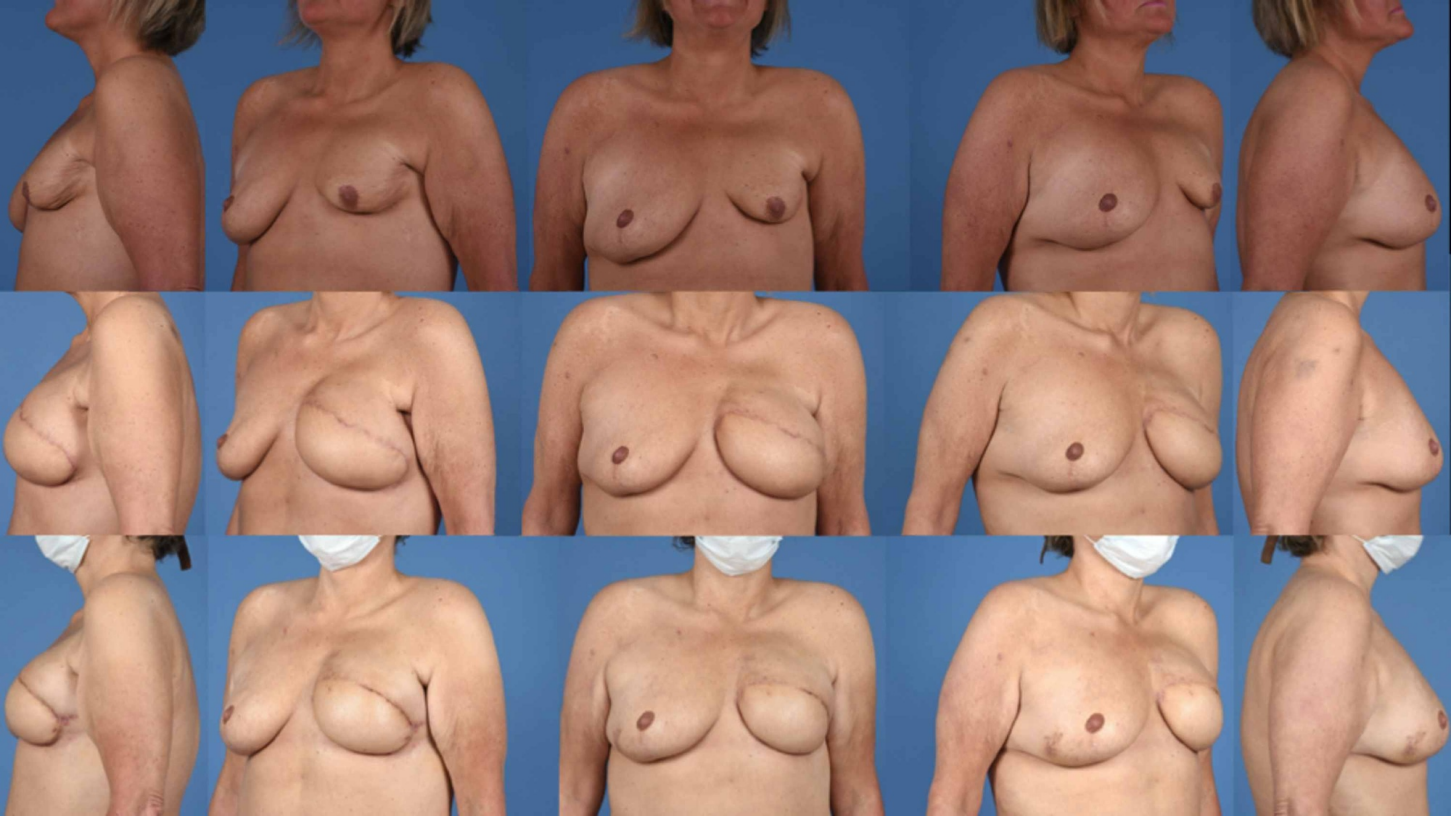
Top panel: Pre-reconstruction images of a 47-year-old female following left total mastectomy. Middle panel: Post-operative images after unilateral free tissue transfer reconstruction from the abdominal donor site. Lower panel: Post-operative images after lateral autologous breast sling.

**Figure 4:**
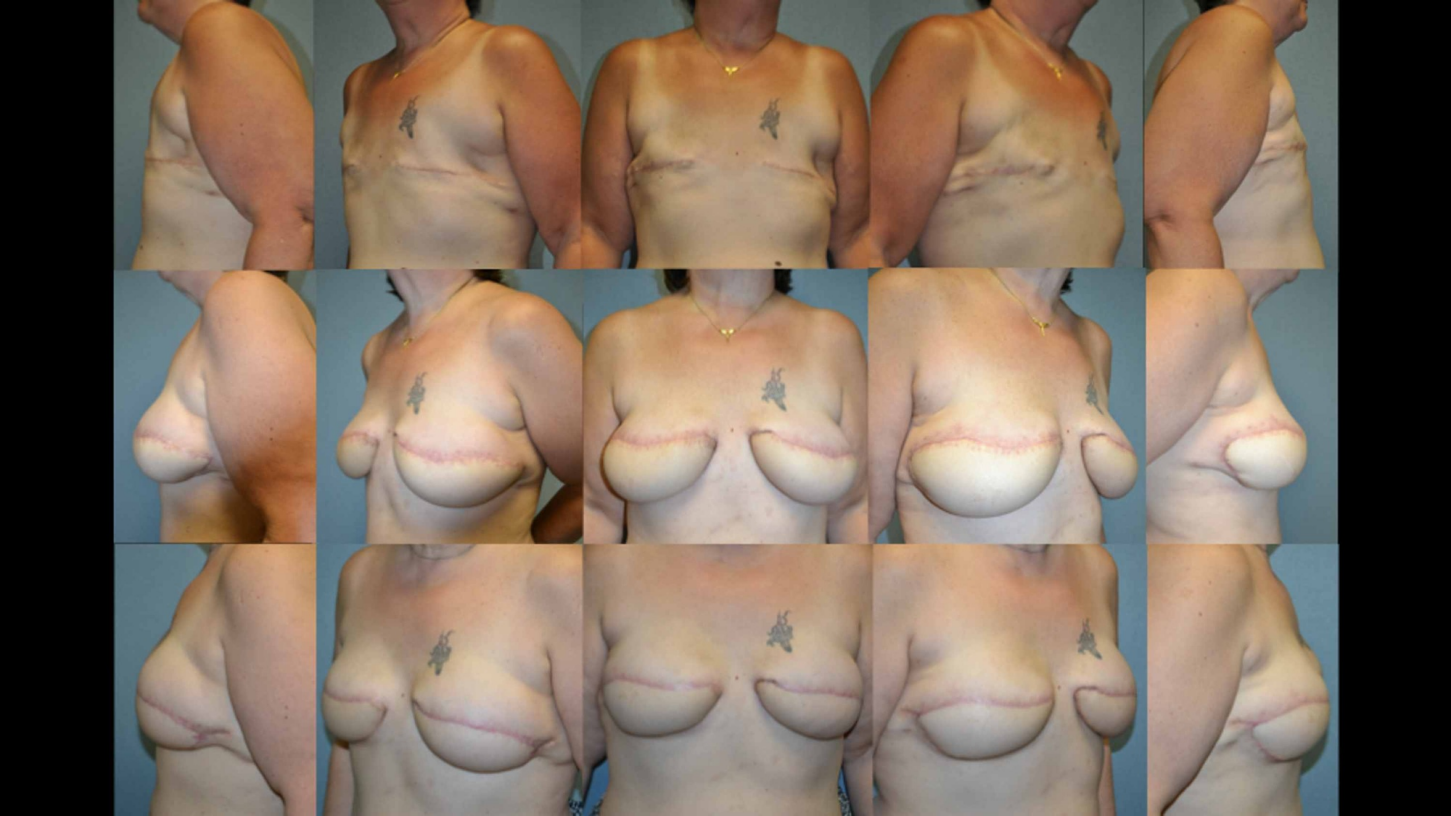
Top panel: Pre-reconstruction images of a 57-year-old female following bilateral total mastectomy. Middle panel: Post-operative images after bilateral free tissue transfer reconstruction from the abdominal donor site. Lower panel: Post-operative images after bilateral lateral autologous breast sling.

Breast reconstruction is complete in 10 of the 14 women included in the study. These 10 women had an average of four total procedures (range 2-5), including the initial abdominal free tissue transfer. The average time to the completion of reconstruction in these women is 13.5 months (range 5-24 months). Eight of these completed reconstructions underwent nipple-areola reconstruction while the other two did not desire this procedure. Four patients continue to be followed in the clinic and have not completed their reconstruction at this time. Figure 3 and Figure 4 are representative outcomes of a unilateral and bilateral lateral autologous flap sling procedure.

## Discussion

Revision for aesthetic improvement of the breast after autologous reconstruction is common, with up to 42% of patients undergoing these secondary procedures [4]. Following mastectomy, remnant excess lateral chest soft tissues can become problematic [2]. Patients frequently complain of discomfort and irritation from their arm rubbing against the lateral chest and redundant axillary skin/adiposity. Further, many patients are unhappy with the appearance of the lateral breast as it transitions to the chest and axilla.

Although autologous and implant breast reconstructions may require secondary revision procedures [4], the wide breast footprint can make a successful tissue transplant less so in the eyes of the patient. Autologous reconstruction techniques transfer new tissue to the chest to create the breast, which is inset to the lateral chest. This may augment the already problematic lateral subunit of the chest in addition to increasing axillary soft tissue excess and disrupting the natural lateral breast contour. Following free tissue transfer breast reconstruction from the abdominal donor site, the most common revision procedures include abdominal scar improvements, liposuction of the transferred flap, and fat grafting of the reconstructed breast [3]. In their review of 150 patients with abdominal free flaps for breast reconstruction, Kim et al. found that 18.7% of patients required liposuction of the lateral breast and 20% received fat grafting of the superomedial pole [3].

Despite their benefits, the techniques commonly employed to revise autologous breast reconstructions have limitations. Lateral breast flap and chest suction-assisted lipectomy result in breast volume reduction but it may not re-shape or re-establish the aesthetic landmarks of the lateral breast. Large volume suctioning is associated with an increased risk of fat necrosis [5]. The central-lateral and lower-lateral breast are most common for liposuction, whereas fat grafting is typically employed for the improvement of the upper-medial and upper-central breast [3]. Dermatolipectomy is typically performed to remove excess lateral chest or breast soft tissues. While this is a safe and effective procedure, it may result in scar extension and blunt the curvature of the lateral breast [2].

The lateral autologous breast sling was developed to add to the techniques available to revise autologous breast reconstructions. The concept originates from techniques noted to improve implant-based reconstructions. Acellular dermal matrix (ADM) slings are commonly used in implant reconstructions to provide additional inferolateral breast implant support and aid in inframammary fold control and definition [6]. Their use in implant-based reconstruction is associated with improved aesthetic results [7]. The new anterior and superior suspension of the lateral flap employed in this technique is similar to ADM use for implants. The flap reorientation creates a more natural and aesthetic breast mound by narrowing the breast, redistributing the lateral breast flap excess to a more medial and superior position, augmenting upper pole fullness, and improving the lateral breast contour.

The lateral breast sling technique addresses the wide breast footprint complaints as well as some of the negative aspects of other revision interventions. By redistributing lateral flap fullness rather than excising it, the lateral breast sling technique addresses multiple common patient complaints. The release of the lateral flap and its repositioning to a more anterosuperior position produces the curving of the lateral breast along the anterior axillary line. In addition, the technique pushes the lateral flap volume medially and superiorly thereby diminishing breast width and establishing an improved lateral breast contour with improved upper pole fullness.

Safety is critical in the use of any adjunct technique. The use of the lateral autologous breast sling in this cohort was demonstrated to be safe with a flap loss, infection, hematoma, and seroma rate of 0%. Women underwent an average of four procedures (Range 2 to 5 procedures) to reach the completion of their reconstruction. Eom et al. in their review of 254 breast reconstructions in 185 patients reported a range of one to 9.4 procedures per breast to complete breast reconstruction [4]. This is consistent with our findings indicating the lateral breast sling does not significantly increase the number of procedures needed to complete breast reconstruction. Additionally, while there is a theoretical risk of vascular compromise with any secondary mobilization of the flap, the lateral breast sling involves dissection remote to the microvascular anastomoses thus minimizing the risk of violation of the pedicle. Moreover, the lateral breast sling technique can be utilized at the time of initial reconstruction with the potential to reduce the need for subsequent procedures.

The lateral breast flap sling technique is by no means without flaws. One such problem is that the technique may not completely resolve upper pole soft tissue deficits. Some of the women included in this study who underwent the sling procedure also had concurrent or staged fat grafting to address the persistent upper pole volume deficits. Breast erythema, fluid collections, and fat necrosis developed in three patients who underwent fat grafting at the time of the lateral breast sling procedure, therefore, the rate of fat necrosis was 21% overall. When the groups were stratified according to body mass index (BMI), 50% of those patients with BMI >30 kg/m^2^ developed fat necrosis as compared to 0% in non-obese patients. Furthermore, compared to other women who underwent concurrent sling and lipofilling, women with fat grafting problems had a higher total fat graft volume. The average volume of fat grafting for patients who developed this complication was 125 cc per breast while the average volume in those without complications was 67 cc per breast (p=0.0081) (Figure 2). Complications associated with higher volumes of fat grafting have been established in the literature. In a retrospective review of 68 patients undergoing fat grafting following autologous breast reconstruction, de Blacam et al. determined that patients who developed complications of fat necrosis, oil cysts, and infection had an average volume of 184 cc of lipofilling per breast in each treatment in comparison to the average of 67 cc of fat graft per breast in each treatment for the total patient population [8]. While one would expect these complications to be associated with higher BMI, previous studies have found no significant relationship between obesity and fat necrosis [9-10]. The patients who developed fat necrosis had BMIs of 31, 31, and 32 kg/m^2^,^ ^which is slightly higher than the average BMI of 29 for the patient group. Given the notable difference in fat graft volume, BMI may be acting as a mediator in our cohort, representing the need for larger volume fat transfer in women with higher BMI. Similarly, there was a concern for infection in two of our patients since they presented with breast erythema. Given their clinical presentation and lack of other signs of infection, this was likely a reflection of fat necrosis, as the cultures from both patients’ fluid collections did not display bacterial growth.

A further consideration regarding the lateral breast sling is whether the flap inset technique employed produced the need for this revision since the flap inset may impact the aesthetics of abdominally-based free flaps. While novel flap inset techniques have been recently described, autologous flaps from the abdomen are most commonly inset in a horizontal or vertical fashion [11]. The aesthetic impact of flap inset was analyzed by Jeong et al., in which 274 patients with unilateral fTRAM or deep inferior epigastric perforator (DIEP) flaps were assessed for aesthetic differences between horizontal and vertical insets [12]. They found that the aesthetic outcomes favored vertical inset with higher scores for symmetry, projection, natural ptosis, and volume in comparison to the contralateral breast. However, it was found that in patients with BMI >25 kg/m^2^, there was no difference between the two groups [7]. Given the average BMI of 29 kg/m^2^ of patients included in this study and the use of a vertical inset pattern, it is unlikely that changes to the inset technique alone would have obviated the need for further revision.

The limitations of this case series include a relatively small sample size as well as the heterogeneity of the patients in terms of age, BMI, and procedures performed. Although unavoidable, reconstruction is not complete in all patients, limiting the ability to draw conclusions from these patients. Similarly, the first patient to undergo concurrent mastectomy, free tissue transfer, and lateral breast sling has not completed reconstruction, making it challenging to assess the long-term effects of this technique at the time of free tissue transfer. The future directions of this study include the continued use of the technique at the time of initial free tissue transfer to determine its effect on total procedures performed as well as aesthetic outcomes.

## Conclusions

The lateral breast flap sling procedure is a novel technique that may serve as an option to the reconstructive surgeon to improve lateral breast contour and improve superior pole volume. The technique may be combined with other autologous breast reconstruction revision procedures to provide a more aesthetically pleasing breast in both unilateral and bilateral mastectomy patients. The use of this technique is associated with a low complication rate and does not significantly increase the total number of reconstructive procedures or duration of reconstruction. The technique may also be useful during initial free tissue transfer.
